# Do NSAIDs suppress polyps in MUTYH-associated polyposis? A case report

**DOI:** 10.1186/1897-4287-9-S1-P36

**Published:** 2011-03-10

**Authors:** Duveen Sturgeon, Alan J Herline, Paul E Wise

**Affiliations:** 1 Department of General Surgery, Vanderbilt University Medical Center, Nashville, Tennessee 37232, USA

## Background

MUTYH-associated polyposis (MAP) is an autosomal recessive disorder that is caused by germline mutations in the base excision repair gene MUTYH. Phenotypic expression is usually that of an attenuated polyposis syndrome with anywhere from 3-100+ polyps and mixed pathology including tubular adenomas, sessile serrated adenomas, and/or hyperplastic polyps. The use of non-steroidal anti-inflammatory drugs (NSAIDs) has been well established as a method of polyp chemoprevention and suppression in familial adenomatous polypsis syndrome (FAP). However, while cyclooxygenase-2 expression has been confirmed in the adenomas, synchronous adenocarcinomas, and healthy mucosa in APC-/MYH+ patients,^1^ it is not well-documented if NSAIDS have any effect on polyp formation in patients with MAP.

## Case report

We report a patient who presented to our clinic with polyposis coli in 2008 after having begun screening colonoscopies at age 44 due to a family history of colorectal cancer (CRC), significant for a brother with colon cancer at age 39 and a sister with polyps/colon cancer at age 71. Given his personal and family history, an attenuated polyposis syndrome was suspected. Genetic testing for APC and MUTYH gene mutations was performed, and he was found to carry biallelic mutations in the MUTYH gene (G382D, Y165C). Polyp progression is well-documented in this patient after his initial colonoscopy in 1994 revealed one small hyperplastic polyp. A total of 7 colonoscopies were done between 1994 and 2006 during which time there were anywhere from 1-6 adenomatous polyps removed. Throughout this time and prior to his first colonoscopy, he utilized scheduled NSAIDs continuously for arthritis (naproxyn sodium followed by celecoxib). In December 2006, celecoxib was discontinued due to a positive stress test and subsequent triple bypass, after which he was placed on only daily 81mg aspirin. The next colonoscopy done 18 months later in 2008 showed multiple polyps throughout the right colon which was a significant change from previous scopes. He was referred to our clinic for evaluation as noted and subsequently underwent a total abdominal colectomy with ileorectal anastomosis.

## Discussion

While his polyp progression may be due to the natural history of his MAP, the chronologic proximity of the advancement of his polyposis phenotype appears more than coincidental. Further studies utilizing NSAIDs for chemoprevention in MAP should be considered.
